# A PRISMA-Driven Systematic Review of Predictive Equations for Assessing Fat and Fat-Free Mass in Healthy Children and Adolescents Using Multicomponent Molecular Models as the Reference Method

**DOI:** 10.1155/2013/148696

**Published:** 2013-06-06

**Authors:** Analiza M. Silva, David A. Fields, Luís B. Sardinha

**Affiliations:** ^1^Exercise and Health Laboratory, CIPER, Fac Motricidade Humana, Univ Tecn Lisboa, 1499-002 Cruz Quebrada, Portugal; ^2^Department of Pediatrics, CMRI Metabolic Research Program, University of Oklahoma Health Sciences Center, Oklahoma City, OK 73104, USA

## Abstract

Simple methods to assess both fat (FM) and fat-free mass (FFM) are required in paediatric populations. Several bioelectrical impedance instruments (BIAs) and anthropometric equations have been developed using different criterion methods (multicomponent models) for assessing FM and FFM. Through childhood, FFM density increases while FFM hydration decreases until reaching adult values. Therefore, multicomponent models should be used as the gold standard method for developing simple techniques because two-compartment models (2C model) rely on the assumed adult values of FFM density and hydration (1.1 g/cm^3^ and 73.2%, respectively). This study will review BIA and/or anthropometric-based equations for assessing body composition in paediatric populations. We reviewed English language articles from MEDLINE (1985–2012) with the selection of predictive equations developed for assessing FM and FFM using three-compartment (3C) and 4C models as criterion. Search terms included children, adolescent, childhood, adolescence, 4C model, 3C model, multicomponent model, equation, prediction, DXA, BIA, resistance, anthropometry, skinfold, FM, and FFM. A total of 14 studies (33 equations) were selected with the majority developed using DXA as the criterion method with a limited number of studies providing cross-validation results. Overall, the selected equations are useful for epidemiological studies, but some concerns still arise on an individual basis.

## 1. Introduction

The rise in the prevalence of childhood obesity [1] has precipitated the need for simple but accurate methods for determining adiposity in paediatric populations. The adolescent years are a period of rapid growth in both the fat (FM) and fat-free mass (FFM) compartments. Despite the recognized importance of measuring body composition in paediatric population, there are a limited number of valid methods that can be used in both clinical and field settings. Most of the simple methods used were developed using the two-compartment (2C) model as the criterion method [2]. The 2C model divides body weight into FM and FFM, relying on assumptions that ignore interindividual variability in the FFM composition, which is the most heterogeneous of the two depots (especially in growing children). Consequently, measured values of FM and FFM are method dependent [3], making accuracy difficult to assess while hindering comparisons across different methods and studies. Multicomponent models, such as 3C and 4C approaches, are robust to inter-individual variability in the composition of the FFM [4]. The model divides body weight into fat, water, mineral, and protein and allows evaluation of several assumed constant relations that are central to 2C models. Although reference data exist for these constants in children from birth to 10 y of age [5], most values were predicted by extrapolating data between infants (6 months) [5] and the 9-year-old reference child [5, 6]. 

The lack of accurate data on body composition further hinders the evaluation of simple field-based techniques such as bioelectrical impedance analysis (BIA) and simple anthropometric measurements. Collectively, these body composition tools are the most commonly used methods in children and adolescents. Variables obtained from BIA and anthropometry are often used as predictors during regression analysis aimed to developed FM and FFM equations based on criterion methods. Given the vast number of BIA and anthropometric-based equations for body composition assessment in children and adolescents, it is difficult to select the most appropriate solution. Therefore, clinicians and health-related professionals need specific and detailed criteria for the appropriate model to select, paying close attention to methodological-, biological- and statistical-related issues that will impact the validity of the body composition value obtained.

### 1.1. Methodological Considerations

In 1992, Wang et al. [7] proposed an interesting system to organize the human body composition, the five-level model. Based on this approach, the human body was characterized in terms of five levels: atomic, molecular, cellular, tissue, and whole body. Most of the methodological research in human body composition analysis has been conducted at the molecular level. Some of the most widely used molecular level models divide body mass into two, three, or four components. As suggested by Wang et al. [8], methods of quantifying these components *in vivo* can be organized using the following general formula:

(1) $$\begin{array}{c} C=f\left(Q\right), \end{array}$$

where *C* represents an unknown component, *Q* a measurable quantity, and *f* a mathematical function relating *Q* to *C* [8]. The mathematical function used in the aforementioned formula can be classified into two types. The first is referred Type I and was developed using a reference method and regression analysis of data to derive the predictive equation [8]. In these cases, a reference method is typically used to measure the unknown component in a group of participants with certain characteristics. The measurable quantity (*Q*, i.e., property and/or the known component), as defined in the general formula, is also estimated. Regression analysis is then used to establish the mathematical function (*f*) and thus, develop the equation that predicts the unknown component [8]. The second type of mathematical function, known as Type II, is based on firmly founded models. These models usually represent proportions or ratios of measurable quantities to components that are assumed constant within and between subjects [8]. Indeed Type II methods are based on assumptions required for their development, and several models have been published. Generally, these models were developed from simultaneous equations, which may include two or more unknown components and/or the measurable property. The less complex Type II methods are based on a 2C model where body mass is divided into FM and FFM, either from hydrometric or densitometric techniques. Type II methods can be described as any of the following combinations. 

(i)  *Two-compartment model*:

(2) $$\begin{array}{c} \text{Body}\;\text{Mass}=\text{fat}+\text{fat-free}\;\text{mass}, \end{array}$$

see [2].

(ii) *Three-compartment model*:

(3) $$\begin{array}{c} \text{Body}\;\text{Mass}=\text{fat}+\text{water}+\text{residual}, \end{array}$$

that is, the sum of protein, minerals, and glycogen [9]:

(4) $$\begin{array}{c} \text{Body}\;\text{Mass}=\text{fat}+\text{bone}\;\text{mineral}+\text{residual}, \end{array}$$

that is, the sum of protein, water, and glycogen [10],

(5) $$\begin{array}{c} \text{Body}\;\text{Mass}=\text{fat}+\text{bone}\;\text{mineral}+\text{lean}\;\text{soft}\;\text{tissue}, \end{array}$$

see [11].

 (iii)  *Four-component model:*

(6) $$\begin{array}{c} \text{Body}\;\text{Mass}=\text{fat}+\text{water}+\text{bone}\;\text{mineral}+\text{residual}, \end{array}$$

that is, the sum of protein, soft tissue minerals and glycogen [12, 13],

(7) $$\begin{array}{c} \text{Body}\;\text{Mass}=\text{fat}+\text{water}+\text{bone}\;\text{mineral}+\text{protein} \end{array}$$

[14, 15].

 (iv)  *Five-component model:*

(8) Body  Mass=fat+water+Bone  mineral+Soft  tissue  mineral+residual,

that is, the sum of protein and glycogen [16].

 (v)  *Six-component model:*

(9) Body  Mass=fat+water+Bone  mineral+Soft  tissue  mineral+protein+glycogen,

see [17].

The densitometric method requires the assessment of body volume (BV), usually estimated by hydrostatic weighing or air displacement plethysmography, serving as the basis for 2C model of body composition analysis. The addition of total-body water (TBW) is allowed for the development of 3C molecular models [9]. The derived 3C model accounted for the variation in subject hydration by adding a TBW estimation using dilution techniques to Behnke's 2C model [2]. On the basis of data available at the time from five chemically analyzed human cadavers, Siri [9] assumed that FFM consisted of two molecular level components, TBW and a combined protein and total mineral [*M*, that is, the sum of soft tissue minerals and bone mineral (*M*_*o*_)] residual component. To complete the model, Siri suggested a constant ratio between mineral and protein of 0.35, as estimated from the five cadavers, with a corresponding density of 1.565 kg/L. 

Dual energy X-ray absorptiometry (DXA) has the advantage of being a 3C model that quantifies total and regional fat mass, lean soft tissue, and bone mineral content. This method assumes that nonosseous tissue consists of two distinct components, fat and lean soft tissue [11]. The lean soft tissue component is the difference between body weight and the sum of fat and bone mineral ash. Fat and lean components are quantified over regions devoid of bone. Typically, the energy source produces photons at two different energy levels, 40 and 70 keV, which pass through tissues and attenuate at rates related to its elemental composition. Bone is rich in highly attenuating minerals, calcium, and phosphorous and is readily distinguished from soft tissues [11]. The measured attenuation of DXA's two main energy peaks is used to estimate each pixel's fraction of fat and lean according to series of physical models [11]. Overall, the DXA method for estimating three components is first, to separate pixels into those with soft tissue only (fat + lean soft tissue) and those with soft tissue + bone mineral, based on the two different photon energies (lower and higher energies, resp.). DXA quantifies FM and FFM with precision [18–21] and provides accurate measures when compared to multicomponent models [22–26]. Indeed, scanning speed and minimal-risk allowed its wide implementation and usage in large multicenter studies, including the National Health and Nutrition Examination Survey [27, 28]. 

The 3C molecular model of Siri [9] can then be extended to a 4C molecular model by adding an estimate of bone mineral by DXA. The 4C model provides the criterion measurements for body composition assessment [29], but its cost, time involvement, poor subject compliance in pediatric populations, and sophisticated technological analysis are impractical for most, if not all nonresearch-based settings. In fact, the 4C model, which divides body mass into FM, water, mineral and protein (and/or residual), is considered the state-of-the art method for assessing body composition as it can accurately account for the variability in the FFM composition [30]. This model involves measurements from different techniques thus allowing the evaluation of several assumed constant relations that are central to 2C models. However, one of the limitations of estimating body fatness from multicomponent models is that combined technical errors occur when each component is separately estimated. While a higher validity is expected with the measurement of more components, there is an associated propagation of measurement errors with the determination of body density (or volume), TBW, and bone mineral. Nevertheless, as long as technical errors are relatively small in each of these components, the cumulative error is also relatively small. Still, when one or more of these components is not precisely measured, the advantages of multicomponent analysis are decreased [29]. Finally, the addition of *in vivo* neutron activation analysis is required to assess soft-tissue minerals and glycogen extending FM estimation from a 4C model to 5C and 6C molecular models.

### 1.2. Biological Considerations

There are many biological conditions where the study of multiple components within the FFM composition is important [30]. Measuring multiple components often reduces the errors of the assumptions in Type II methods specifically in pediatric populations that can vary substantially the contribution of main FFM components due to growth and maturation. As previously stated, 2C models, use either hydrometric or densitometric techniques and are based upon constants that came from a few adult human cadaver dissections, animal data, and indirect estimates of FFM in human subjects [9, 31, 32]. This approach is less accurate in children because of potential changes in the various assumptions of 2C models during growth and maturation, such as changes in the density and hydration of the FFM [10]. Therefore, the 4C model is robust to interindividual variability in the FFM and is the “gold standard” in pediatric populations [33]. However, multicomponent models are costly, time consuming, and impractical for most settings. For example, to assess FM, a typical 4C model study requires many hours for completion, normally starting with isotope dilution for TBW and measurement of body mass. Then, underwater weighing or air displacement plethysmography and DXA techniques, respectively, for body volume and bone mineral assessment are needed. Two measurable quantities, TBW and bone mineral along with two properties, body volume and mass, are required to calculate FM.

An alternative solution in overcoming the lack of accuracy using less complex techniques based upon 2C models is the use of age- and sex-specific constants derived from pediatric populations. Hydrometry and densitometry are two techniques widely used to assess pediatric body composition due to their ease of application, but their validity depends on the accuracy of age- and sex-specific constant values for FFM hydration or density. Since 1980, these constants have relied upon empirical data from Fomon et al. [5] that published body composition values for a reference child starting at birth going to 10 y, with most of the values extrapolated from other data [34]. Lohman [10] provided similar reference data for pediatric ages based on simultaneous measurements of TBW, body density, and forearm bone mineral density [34, 35]. Simulations for adolescents were also reported by Haschke [6]. Based on these studies and extrapolations, Table 1 presents sex- and age-specific constants for conversion of body density, water, and mineral to percent fat in children and adolescents.

Recently, Wells et al. [33] reported reference data for the hydration and density of the FFM and developed prediction equations on the basis of age, sex, and body mass index standard deviations using the 4C measures obtained in a large, healthy sample of children and adolescents aged 4–23 years.  Table 2 represents the median values proposed by the authors for hydration, density, and constants using the LMS (lambda-mu-sigma) method. Using these values it is possible to substitute C1 and C2 constants in Siri's [9] equation, thus, improving the accuracy of densitometric techniques in estimating FM of a healthy pediatric population. 

In addition, the age- and sex specific constants for FFM hydration presented in Table 2 can be used to improve the accuracy of hydrometric methods known to be based on the following stable relationship:

(10) $$\begin{array}{c} \text{FFM}\left(\text{kg}\right)={\text{FFM}}_{\text{TBW}}*\text{TBW}\left(\text{kg}\right), \end{array}$$

where FFM_TBW_ stands for fat-free mass hydration based on the age- and sex-specific constants and TBW for total body water. This equation can be rearranged to

(11) $$\begin{array}{c} \%\text{FM}=\left(\frac{\text{FM}}{\text{BM}}\right)*100, \end{array}$$

where FM is assessed from subtracting FFM from body mass (BM). It is important to emphasize if adult values are used rather than the proposed age- and sex-specific constants in the estimation of FM from densitometric and hydrometric methods, an over- and underestimation of adiposity is expected, respectively. In fact, Siri's 3C model by including both TBW and density is a valid model for determining FM during growth, overcoming the limitations of measuring total body density alone. Hence, the combination of body density and body water has become the most practical multicomponent approach to body composition assessment in growing children [10]. With the development of improved body water procedures through deuterium dilution [34, 36, 37], this approach has offered better estimates of FM and FFM in this population. 

Though the use of age- and sex-specific constants improves the accuracy of 2C models in assessing FM and FFM in children, simpler field-based methods are still needed. Therefore, if the goal is to develop field-based techniques to predict body composition, multicomponent models should be used as the preferred criterion method. Therefore, the accuracy of anthropometry and BIA-based equations are dependent in part on the accuracy of the criterion variable for measuring FM and FFM but also on the statistical procedures used to develop these Type I functions.

### 1.3. Statistical Approach for Developing Predictive Equations

In this section, we will review the most common methods used to developed predictive models, that is, Type I functions for assessing body composition with regression analysis, the most widely used method for their development. Briefly, predictor variables that show the highest correlation with the response variable are chosen to yield the maximum *R* ^2^ (representing the proportion of the total variance in the response variable that is explained by the predictors in a given equation) [38]. Then, a second significant variable is added to the model with the amount of shared variance increasing the *R* ^2^. The procedure is repeated to achieve the best combination of predictor variables until the inclusion of any variable no longer improves (i.e., significantly) the *R* ^2^ [38]. 

Another concern when developing predictive equations is multicollinearity, a condition where independent variables are strongly correlated with each other. Therefore, if too many variables are included as predictors in a given equation, the probability of multi-collinearity is increased. The variance inflation factor, defined as 1/(1 − *R* ^2^), can be calculated to detect multi-collinearity. To reduce the number of equations generated and the chance of multi-collinearity, the elimination of predictor variables with the lowest correlation with the reference method should be performed [38]. Additionally, to assure the appropriate number of predictors in a specific equation, Mallows' Cp statistic index [39] should be used. According to Sun and Chumlea [38], the equation with the minimum Cp will have the maximum *R* ^2^ and minimum root mean square error (RMSE) values, and as expected, a reduced bias and multi-collinearity. In the development of the regression model, the larger the *R* ^2^ the better the equation fits the data, whereas the precision of the model is evaluated by the RMSE. The RMSE is calculated as the square root of the sum of squared differences between the predicted and the observed values divided by the total number of observations minus the number of parameters [38] as follows:

(12) $$\begin{array}{c} \text{RMSE}=\sqrt{\frac{\sum {\left(\text{observed}-\text{predicted}\right)}^{2}}{\left(n-p-1\right)}}, \end{array}$$

where *n* is the number of observations, and *p* is the number of predictor variables. The RMSE should be standardized for the mean value of the criterion method. This procedure is called the coefficient of variation (CV), a standardized value that is useful in comparing predictive equations with different response variables and different units [38]. 

Generally, there are specific selection criteria that should be used for testing the accuracy of new predictive Type I functions. One of the first criteria is the validity of the reference method because of its inherent error of measurement, which dose not allow for perfect criterion scores. According to Sun and Chumlea [38], other performance indicators include sample size, the ratio of sample size to the number of predictor variables, size of the coefficient of correlation (*R*), *R* ^2^, RMSE, and the CV for the equation [38]. To measure the increase in sample size necessary to offset the loss of precision, the ratio between the variance of prediction error and the variance of criterion value should be calculated [40]. For example, a sample of 100 participants is required to achieve a significant 1% increase in *R* ^2^ precision or accuracy of a predictive equation with a statistical power of 90% [38]. An additional procedure to assess the generalizability of predictive equations is the cross-validation of developed models. To test the performance of a predictive equation in cross-validation studies, the pure error (PE) is used. The parameter is calculated as the square root of the sum of squared differences between the observed and the predicted values divided by the number of subjects in the cross-validation sample [38] as follows:

(13) $$\begin{array}{c} \text{PE}=\sqrt{\frac{\sum {\left(\ddot{Y}-Y\right)}^{2}}{n}}, \end{array}$$

where $\ddot{Y}$ are the predicted values, *Y* are the observed values, and *n* is the number of subjects. While smaller RSME values indicate a greater precision in the development of a predictive equation, a reduced PE points to a better accuracy of the equation when applied to an independent sample. The cross-validation procedure involves the application of the developed model in another sample from the population. Usually 2/3 of the sample is used for developing a prediction equation, and 1/3 is used to cross-validate the model though other procedures can be used, such as the Jackknife method and the prediction of the sum of squares (PRESS) [41, 42]. To test the accuracy of an equation when applied to the cross-validation sample, the following parameters should be analyzed: size of the *R* ^2^, PE, and the potential for bias (mean difference between methods). Further, though less used, the concordance correlation coefficient (CCC) proposed by Lin [43], should be examined as it represents a measure of accuracy by indicating a bias correction factor that quantifies how far the best fit line deviates from the 45° line through the origin, and a measure of precision that specifies how far each observation deviates from the best-fit line. Also, for testing the performance of the newly developed equation in the cross-validation group, the agreement between methods should also be examine by analyzing the 95% limits of agreement, as proposed by Bland and Altman [44], which tests the potential for bias across the range of fatness or leanness. This is calculated by the differences of the methods (*y*-axis) and the mean of the methods (*x*-axis) (as proposed by Bland and Altman [44]). Instead, the residuals of the regression between methods with the criterion (in abscissas) have also been reported [45]. The presence of a trend between the differences and the mean of the methods is determined by using the coefficient of correlation (or instead by observing the homoscedasticity of the residuals); this is to say a significant correlation between the *x*- and *y*-axis indicates bias across the range of fatness.

### 1.4. Objectives

The present study aims to review all the available BIA and/or anthropometric-based equations published between 1985 and 2012 for body composition assessment developed using 3C and 4C models in the paediatric population.

## 2. Methods

An extensive literature review was conducted, according to the guidelines proposed at the PRISMA statement [46], to select predictive equations for body composition estimation in a paediatric population. MEDLINE database (OVID, PubMed) and Thomson Reuters Web of Knowledge platform were searched for English language articles published in peer-reviewed journals since 1985 with the last search run on December 11, 2012. The keyword search terms included: children, adolescent, childhood, adolescence, four-compartment model, three-compartment model, multicomponent model, equation, prediction, dual-energy X-ray absorptiometry, bioelectrical impedance analysis, resistance, anthropometry, skinfold, fat, and fat-free mass. The following characteristics and criteria were used: (1) participants were healthy children and adolescents; (2) the predictor variables were based on BIA and/or anthropometry; (3) the 3C and 4C models were used as the criterion methods; (4) relative or absolute FM and FFM were assessed; (5) detailed description of the statistical methods used to formulate the equations was provided. For the identification of studies, the process included the following steps: screen of the identified records; examination of the full text of potentially relevant studies; and application of the eligibility criteria to select the included studies. For assessing eligibility, studies were screened independently in an unblinded standardized manner by the primary author, whereas the secondary author examined a small sample of them.

## 3. Results

**Figure 1:**
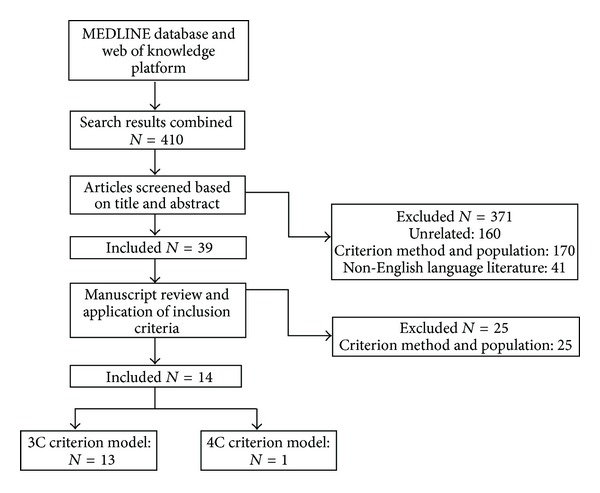
Flow diagram of study selection [46].

Our search provided a total of 410 citations. Of these, 371 studies were discarded because after reviewing the title and abstract, it appeared that these papers clearly did not meet the criteria. The full text of the remaining 39 citations was examined in more details. A total of 25 studies did not meet the inclusion criteria described in Section 2; therefore, a total of 14 studies involving 33 equations were identified for paper. A flow diagram is illustrated in Figure 1 to describe the number of studies screened, assessed for eligibility, and included in the paper, along with reasons for exclusions at each stage.

A detailed description of the selected equations is presented in Tables 3 and 4, including the characteristics of the study sample, the response and the predictor variables, the criterion models, and the statistical methods used to validate and formulate the equations.

The studies summarized in Table 3 presented *R* ^2^ values for relative and absolute FM ranging from 0.85 to 0.93 and from 0.55 to 0.96, respectively, with RMSEs ranging from 2.60 to 3.40% for %FM and from 0.94 to 4.29 kg for absolute FM. Values of *R* ^2^ > 0.94 and RMSE ranging from 1.0 to 2.1 kg were found for FFM estimation. In Table 4, equations developed using a 4C model as the reference method [47] yielded *R* ^2^ that ranged from 0.76 to 0.82 with RMSE ranging from 3.6 to 3.8%. The CVs were not available for the majority of the equations. Overall, DXA was used as the reference method to estimate FM [48–53], %FM [54, 55], and FFM [56–58].

Among the 33 equations presented in Tables 3 and 4, only 7 were cross-validated [48, 52, 53, 56, 58, 59]. Only 2 studies examined the PEs [56, 58] in estimating FFM, ranging from 1.2 to 1.5 kg. During the cross-validation analysis, *R* ^2^ values ranged from 0.80 to 0.92 for absolute FM with no available information for relative FM. Cross-validation of FFM reported in one study [56] showed an *R* ^2^ value of 0.95 whereas another study provided values for the CV [58] that ranged from 5 to 6%. None of the above studies examined the CCC, whereas agreement between methods was only included in 3 studies [48, 53, 56]. The smaller 95% confidence intervals for absolute FM were found for Dezenberg equations (−0.3 to 0.1 kg), while Huang equation ranged from −5.7 to 6.4 kg. For Clasey equation, FFM limits of agreement ranged from −2.4 to 2.5 kg. For all the cross-validation equations, the difference between the predictive and the reference methods showed values closed to 0, indicating a reduced bias in the cross-validation sample of the aforementioned studies. 

## 4. Discussion

A total of 33 BIA and anthropometric-based equations for assessing body composition using multicomponent models as the reference method met the criteria and were selected and reviewed. Overall, these models provided an acceptable accuracy to be used in epidemiological studies. Generally, BIA-based equations were developed for FFM estimates, whereas anthropometric-based models were developed for FM estimates. 

Several equations were developed for ages below 14 years while few published equations covered a larger broad of ages, respectively, 3 to 18 years [49, 50] and 6 to 17 y [55, 58]. The studies of Ellis et al. [49, 50] likewise presented the largest and ethnically diverse sample, including Caucasian, Hispanics, and Blacks, though the male equations only explained ~60% of the variance in the reference method. 

Also, of note is the absence of including a multi-collinearity analysis in the majority of the selected equations with the exception of the predictive model proposed by Morrison et al. [58]. A limited number of studies included a standardized value (CV) for the RMSEs [49, 50, 58, 59], a useful parameter for comparing predictive equations with different response variables and units.

Another important finding is the small number of studies that actually reported the cross-validation of newly proposed models [48, 52, 53, 56, 58, 59]. This is a major flaw in the ability to generalize the predictive model as it establishes whether the equation was accurate to sample-specific variations. In this regard, it is important to highlight the equation developed by Clasey et al. [56] for FFM estimation using BIA in a large sample of Caucasian children aged 5–11.9 year. The cross-validation sample used by the authors [56] comprising ~80 children explained FFM variability from the criterion method by 95%. The few studies that reported agreement between the proposed equation and the criterion method when applied to a cross-validation sample indicated that limits of agreement are relatively larger which may limit the accuracy of the models at an individual level, even though the mean bias was small. Additionally, none of the studies that included a cross-validation sample analysed the concordance correlation coefficient (CCC) proposed by Lin [43], as it represents in the same calculation a measure of accuracy and precision of the proposed methodology in relation to the reference technique.

Most of the studies presented in Table 3 were developed using DXA as the criterion method either to estimate FM [48–53], %FM [54, 55], or FFM [56–58] using different instruments, models, and scan modes. The validity of the response variable, that is, the criterion method, is determinant for developing appropriate equations based on BIA and/or anthropometry. Therefore, the usefulness of DXA as the reference method for the development of several proposed equations needs to be addressed, in particular some advantages and shortcomings of this technique to assess body composition in pediatric populations. Recently, Toombs et al. [60] pointed out that DXA technological advances demonstrated a good precision, large availability, and low radiation dose, highlighting DXA as a convenient and useful diagnostic tool for body composition assessment. These authors also concluded that DXA technology can be improved if the uncertainties associated with the trueness of DXA body composition measurements are addressed by conducting more validation studies for testing different DXA systems against *in vivo *methods such as neutron activation analysis and the 4C model [60]. Systematic variations between devices and software versions have been reported previously [61, 62]. Therefore, DXA systems are not interchangeable and generalizability of predictive equations generated by different densitometers, software, and/or scan mode is still unknown. Further research is required for addressing methodological issues related to the validity of this technique, especially if it is used as a criterion method for developing alternative solutions for body composition assessment. 

It is recognized that 4C models are the best approach in pediatric populations for developing and cross-validating new body composition methods. Though other studies [63, 64] included children and adolescents in the prediction of bedside techniques using a 4C model as the criterion method, only Slaughter et al. [47] proposed solutions specifically developed for a healthy pediatric population ranging in age, maturation status, gender, ethnicity, and adiposity level. This model included bone mineral assessment from a single photon absorptiometry, and the impact of this estimation on the accuracy of those models is still unknown. Sun et al. [63] and Horlick et al. [64] also developed BIA-based equations for assessing FFM using a 4C model as the criterion method. However, we did not include these equations since a wide range of age was found for Sun et al.'s proposed models (12–94 years) [63], whereas Horlick et al. [64] included HIV-infected children along with healthy children during model development. It is important to address that multicomponent molecular models do not rely upon major assumptions regarding proportions of the FFM density or hydration which are the cornerstone of 2C models. However the use of 3C and 4C models is highly expensive, and laborious which disables its implementation in most laboratories. Though the precision of multicomponent models may be affected by the propagation of measurement error related to the need of assessing several techniques, reliability of 3C and 4C models is not compromised if technical errors are relatively small [4].

## 5. Conclusion

In this paper, BIA and anthropometric-based equations developed against multicomponent models for estimating FM and FFM in children and adolescents were examined. Very few equations included a cross-validation sample, and future research efforts should include this procedure for newly proposed models to eliminate the least accurate and precise rather than to continue developing new equations.

We identified 33 prediction equations that are acceptable alternatives for epidemiological/clinical settings. The predictive equations of Slaughter, developed against a 4C model, used a wide and diverse sample ranging in age, maturation status, ethnicity, gender, and adiposity levels and should, therefore, be recommended as a feasible and valid alternative for assessing body composition in paediatric populations. 

Multicomponent models, specifically the 4C model, can account for potential effects of age, sex, and ethnicity differences in the FFM density and composition when used as the criterion method nevertheless residual differences can occur. Therefore, specific BIA and/or anthropometric models for clearly defined ages, gender, and ethnic groups of children and adolescents are required using a 4C model as the criterion method. 

Finally, future research studies should employ multicomponent models to accurately address the dynamic changes in paediatric body composition using, as predictors, whole body measures.

## Figures and Tables

**Table 1:** Age- and sex-specific constants for conversion of body density, water, and mineral to %FM in children and youth.

Age (years)		Females			Males	
	*D*_FFM_	*C*1	*C*2	*D*_FFM_	*C*1	*C*2
7–9	1.079	5.451	5.052	1.081	5.400	4.996
9–11	1.082	5.376	4.968	1.084	5.327	4.914
11–13	1.086	5.279	4.861	1.087	5.255	4.835
13–15	1.092	5.141	4.708	1.094	5.098	4.660
15–17	1.094	5.098	4.660	1.096	5.055	4.612
17–20	1.095	5.076	4.636	1.099	5.002	4.554
20–25	1.096	5.055	4.612	1.100	4.971	4.519

%FM: percent fat mass; Db: body density; *D*_FFM_: fat-free mass density; *C*1: constant 1; *C*2: constant 2.

*Calculation of percent fat mass (%FM) using age- and sex-specific values for the density of the FFM: %FM = [(*C*1/Db) −  *C*2] ∗ 100, where Db represents body density. Adapted from Lohman [10].

**Table 2:** Median values for hydration, density, and constants (*C*1 and *C*2) for the paediatric version of Siri's (11) equation, obtained by using the LMS (lambda-mu-sigma) method*.

Age	Males				Females			
	Hydration %	Density kg/L	*C*1	*C*2	Hydration %	Density kg/L	*C*1	*C*2
5 y	76.5	1.0827	5.36	4.95	76.7	1.0837	5.33	4.92
6 y	76.3	1.0844	5.32	4.90	76.1	1.0865	5.27	4.85
7 y	76.1	1.0861	5.28	4.86	75.5	1.0887	5.22	4.79
8 y	75.9	1.0877	5.24	4.82	75.2	1.0900	5.19	4.76
9 y	75.7	1.0889	5.21	4.79	75.1	1.0909	5.17	4.74
10 y	75.5	1.0900	5.19	4.76	75.0	1.0916	5.15	4.72
11 y	75.3	1.0911	5.16	4.73	75.0	1.0924	5.13	4.70
12 y	75.2	1.0917	5.15	4.72	74.9	1.0937	5.10	4.67
13 y	75.0	1.0920	5.14	4.71	74.6	1.0954	5.07	4.63
14 y	74.8	1.0927	5.13	4.69	74.4	1.0975	5.02	4.58
15 y	74.4	1.0942	5.09	4.66	74.1	1.0996	4.98	4.53
16 y	74.0	1.0960	5.05	4.61	73.8	1.1011	4.95	4.49
17 y	73.7	1.0978	5.02	4.57	73.7	1.1020	4.93	4.47
18 y	73.5	1.0991	4.99	4.54	73.6	1.1027	4.92	4.46
19 y	73.4	1.1000	4.97	4.52	73.6	1.1031	4.91	4.45
20 y	73.3	1.1006	4.96	4.51	73.6	1.1035	4.90	4.44

∗*C*1 is calculated as (*D*_FFM_ ∗ *D*_FM_)/(*D*_FFM_ − *D*_FM_), and *C*2 is calculated as D_FFM_ /(*D*_FFM_ − *D*_FM_); *D*_FFM_ and *D*_FM_ represent fat-free mass density and fat mass density, respectively. % fat mass is calculated as [(*C*1/Db) – *C*2] ∗ 100, where Db is measured body density. Adapted from Wells et al. [33].

**Table 3:** Summary of predictive equations for children and adolescents based on a three-compartmental model.

					Development					Cross-validation								
Author	Sex	Ethnic group	Age Y	*N*	Criterion method	*R* ^2^	RMSE	CV (%)	Equations and predictor variables	*N*	Age	*R* ^2^	PE	CV	CCC	Bias	Agreement	
																	Limits	Trend
Houtkooper et al. [65]	M	Cauc	10–14	53	UWW, Deut Dilut^1^	0.85	3.3%	NA	%FM = −0.235 ∗ S, cm^2^/*R* + 0.252 ∗ Abdcirc + 0.281 ∗ (sum: Tric, Abd, Thigh SKF) − 0.044	NA	NA	NA	NA	NA	NA	NA	NA	NA
	F			41														

Houtkooper et al. [65]	M	Cauc	10–14	53	UWW, Deut Dilut^1^	0.94	1.9 kg	NA	FFM (kg) = 0.713 ∗ S, cm^2^/*R* + 0.15 ∗ (chest circ) + 0.493 ∗ (hip Skeletal width) + 0.121 ∗ Re − 21.41	NA	NA	NA	NA	NA	NA	NA	NA	NA
	F			41														

Houtkooper et al. [59]	M and F	Cauc	10–14	157^2^	UWW, Deut Dilut^1^	0.95	2.1 kg	5.1	FFM (kg) = 0.61 ∗ S, cm^2^/*R* + 0.25 ∗ *W* + 1.31	25	10.5–14.4	0.95	NA	NA	NA	1.7	NA	NA

Goran et al. [51]	M	Cauc	4–9	49	DXA Lunar DPX-L densitometer	0.91	0.94 kg	NA	FM (kg) = 0.16 ∗ Sub SKF + 0.33 ∗ *W* + 0.11 ∗ Tric SKF − 0.16 ∗ S, cm^2^/*R* − 0.43 ∗ Sex − 2.4	NA	NA	NA	NA	NA	NA	NA	NA	NA
	F			49		0.88	1.05 kg	NA	FM (kg) = 0.18 ∗ *W* + 0.23 ∗ Sub SKF + 0.23 ∗ Tric SKF − 3.0									

Ellis [49]	M	Cauc	3–18	145	DXA Hologic QDR 2000	0.57	3.56 kg	31.7	FM (kg) = 0.534 ∗ *W* − 1.59 ∗ age + 3.03	NA	NA	NA	NA	NA	NA	NA	NA	NA
		Black		78		0.62	4.29 kg	36.1	FM (kg) = 0.594 ∗ *W* − 0.381 ∗ S, cm + 36.0	NA	NA	NA	NA	NA	NA	NA	NA	NA
		Hispanic		74		0.55	3.71 kg	25.7	FM (kg) = 0.591 ∗ *W* − 1.82 ∗ Age + 3.36	NA	NA	NA	NA	NA	NA	NA	NA	NA

Ellis et al. [50]	F	Cauc	3–18	141	DXA Hologic QDR 2000	0.93	1.09 kg	9.7	FM (kg) = 0.642 ∗ *W* − 0.12 ∗ S, cm − 0.606 ∗ Age + 8.98	NA	NA	NA	NA	NA	NA	NA	NA	NA
		Black		104		0.96	2.44 kg	16.7	FM (kg) = 0.653 ∗ *W* − 0.163 ∗ S, cm − 0.298 ∗ Age + 10.7	NA	NA	NA	NA	NA	NA	NA	NA	NA
		Hispanic		68		0.95	2.45 kg	15.1	FM (kg) = 0.677 ∗ *W* − 0.217 ∗ S, cm + 15.5	NA	NA	NA	NA	NA	NA	NA	NA	NA

de Lorenzo et al. [57]	M	NA	7.7–13	20	DXA Lunar DPX	0.96	1.0 kg	NA	FFM = 2.330 + 0.588 ∗ S, cm^2^/*R* + 0.211 ∗ *W*	NA	NA	NA	NA	NA	NA	NA	NA	NA
	F			15	densitometer													

Dezenberg et al. [48]	M and F	Cauc and Black	4–10.9	135	DXA Lunar DPX-L	0.95	0.5 kg	NA	FM (kg) = 0.332 ∗ *W* + 0.263 ∗ Tric SKF + 0.76 ∗ Sex + 0.704 ∗ Ethnicity − 8.004	67	4–10.9	0.92	NA	NA	NA	−0.11	0.1–(−0.3)	0.19

Morrison et al. [58]	F	Cauc	6–17	65	Hologic QDR-1000/W	0.99	1.14 kg	3.6	FFM (kg) = 1.07 + 0.37 ∗ S, cm^2^/*R* 0.17 ∗ Tric SKF + 0.47 ∗ *W*	20	9.3 ± 0.6	NA	1.5	6	NA	NA	NA	NA
		Black		61		0.99	1.95 kg	4.7	FFM (kg) = −8.78 + 0.78 ∗ S, cm^2^/*R* + 0.10 ∗ Rc + 0.18 ∗ *W*	20	9.2 ± 0.5	NA	1.2	5	NA	NA	NA	NA

Bray et al. [54]	M and F	Cauc and Black	10–12	129	DXA Hologic QDR 2000	0.91	3.1%	NA	%FM = 7.26 + 0.77 ∗ Bic SKF + 0.36 ∗ calf SKF + 0.25 ∗ Thigh SKF	NA	NA	NA	NA	NA	NA	NA	NA	NA
						0.89	3.4%	NA	%FM = 9.02 + 1.09 ∗ Bic SKF + 0.42 ∗ Calf SKF	NA	NA	NA	NA	NA	NA	NA	NA	NA

Clasey et al. [56]	M	Cauc	5–11.9	203	DXALunar DPX-IQ	0.95	1.4 kg	NA	FFM (kg) = −7.655 + 0.297 ∗ S, cm + 0.125 ∗ *W* – 0.0174 ∗ Imp	38 M	5–11.9	0.95	1.2 kg	NA	NA	0.0	2.5–(−2.4)	NS
	F			158	(GE/Lunar)					37 F								

Flavel et al. [55]	M	Cauc	6–17	37	DXA Lunar Prodigy	0.91	3.0%	NA	%FM = 1.09 + 0.63 ∗ Tri SKF + 0.36 ∗ Thigh SKF + 0.50 Supraspinale SKF – 0.16 ∗ Abd SKF + 0.33 Bic SKF	NA	NA	NA	NA	NA	NA	NA	NA	NA
	F			33		0.93	2.60%	NA	%FM = 11.03 + 0.93 ∗ BMI + 0.30 ∗ Waist Girth − 0.24 ∗ S, cm + 0.48 ∗ Calf SKF + 0.07 ∗ Hip Girth	NA	NA	NA	NA	NA	NA	NA	NA	NA

Huang et al. [53]	M and F	Latino	7–13	64	DXA Hologic QDR 4500W	0.92	NA	NA	FM (kg) = 0.665 ∗ *W* − 1.606 ∗ Age − 1.882 ∗ Sex (0 = girl; 1 = boy) + 3.330	32	7–13	0.92	NA	NA	NA	0.36	6.4–(−5.7)	NS

Hoffman et al. [52]	M	Mixed ethnicity	9.8 ± 1.3	48	DXA Hologic QDR 4500A	0.78	1.2 kg	NA	FM (kg) = 6.371 + 0.488 ∗ *W* + 0.128 ∗ Tric SKF − 1.138 ∗ S, cm + 0.645 ∗ Sex − 0.188 ∗ Age	12	10.1 ± 1.5	0.80	NA	NA	NA	0.09	NA	NA
	F			67						18								

NA: not available; UWW: underwater weighing; DXA: dual-energy X-ray absorptiometry; Cauc: caucasians; SKF: skinfold; NS: nonsignificant; Bic SKF: bicipital skinfold; Tric SKF: tricipital skinfold; Supil SKF: Suprailiac skinfold; Sub SKF: subscapular skinfold; Abd SKF: abdominal skinfold; M: male; F: female; Deut Dilut: deuterium dilution; *R* ^2^: coefficient of determination; RMSE: root of mean square error; CV: coefficient of variation; CCC: concordance correlation coefficient; PE: pure error; BMI: body mass index; *W*: weight; FM: fat mass; FFM: fat-free mass; S: stature; *R*: resistance; Rc: reactance; Imp: impedance; abdcirc: abdominal circumference.

^1^% Fat = {(2.057/Db) − 0.786 ∗ *W* − 1.286} ∗ 100, where Db: body density; *W*: total body water [9].

^2^% Final equation was obtained from combining the development and cross-validation samples [59].

**Table 4:** Summary of predictive equations for children and adolescents published based on a four-compartmental model.

					Development					Cross-validation								
Author	Sex	Ethnic group	Age Y	*N*	Method and criterion model	*R* ^2^	RMSE	CV (%)	Equations and predictor variables	*N*	Age	*R* ^2^	PE	CV (%)	CCC	Bias	Agreement	
																	Limits	Trend
Slaughter et al. [47]	M	Cauc and Black	8–18*	174	UWW, Deut Dilut, SPA^1^	0.78	3.8%	NA	%FM = 0.735 ∗ (Sum: Tric and Calf SKF > 35 mm) + 1.0	NA	NA	NA	NA	NA	NA	NA	NA	NA
	F			136		0.78	3.8%	NA	%FM = 0.610 ∗ (Sum: Tric and Calf SKF > 35 mm) + 5.1	NA	NA	NA	NA	NA	NA	NA	NA	NA

Leaner children and adolescentes (Sum: Tric + Sub SKF < 35 mm)																		

Slaughter et al. [47]	M	Cauc	Prepub	50	UWW, Deut Dilut, SPA^1^	0.80	3.6%	NA	%FM = 1.21 ∗ (Sum: Tric and Sub SKF < 35 mm) − 0.008 ∗ (Sum: Tric and Sub SKF < 35 mm)^2^ − 1.7	NA	NA	NA	NA	NA	NA	NA	NA	NA
		Black				0.80	3.6%	NA	%FM = 1.21 ∗ (Sum: Tric and Sub SKF < 35 mm) − 0.008 ∗ (Sum: Tric and Sub SKF < 35 mm)^2^ − 3.5	NA	NA	NA	NA	NA	NA	NA	NA	NA
		Cauc	Pub	30		0.82	3.6%	NA	%FM = 1.21 ∗ (Sum: Tric and Sub SKF < 35 mm) − 0.008 ∗ (Sum: Tric and Sub SKF < 35 mm)^2^ − 3.4	NA	NA	NA	NA	NA	NA	NA	NA	NA
		Black				0.82	3.6%	NA	%FM = 1.21 ∗ (Sum: Tric and Sub SKF < 35 mm) − 0.008 ∗ (Sum: Tric and Sub SKF < 35 mm)^2^ − 5.2	NA	NA	NA	NA	NA	NA	NA	NA	NA
		Cauc	Pospub	58 36		0.76	3.6%	NA	%FM = 1.21 ∗ (Sum: Tric and Sub SKF < 35 mm) − 0.008 ∗ (Sum: Tric and Sub SKF < 35 mm)^2^ − 5.5	NA	NA	NA	NA	NA	NA	NA	NA	NA
		Black				0.76	3.6%	NA	%FM = 1.21 ∗ (Sum: Tric and Sub SKF < 35 mm) − 0.008 ∗ (Sum: Tric and Sub SKF < 35 mm)^2^ − 6.8	NA	NA	NA	NA	NA	NA	NA	NA	NA
	F	Cauc and Black		136	UWW, Deut Dilut, SPA^1^	0.80	3.9%	NA	%FM = 1.33 ∗ (Sum: Tric and Sub SKF < 35 mm) − 0.013 ∗ (Sum: Tric and Sub SKF < 35 mm)^2^ − 2.5	NA	NA	NA	NA	NA	NA	NA	NA	NA

Fatter children and adolescentes (Sum: Tric + Sub SKF > 35 mm)																		

Slaughter et al. [47]	M	Cauc and Black		174	UWW, Deut Dilut, SPA^1^	0.80	3.6	NA	%FM = 0.783 ∗ (Sum: Tric and Sub SKF > 35 mm) + 1.6	NA	NA	NA	NA	NA	NA	NA	NA	NA
	F			136		0.80	3.9	NA	%FM = 0.546 ∗ (Sum: Tric and Sub SKF > 35 mm) + 9.7	NA	NA	NA	NA	NA	NA	NA	NA	NA

NA: not available; UWW: underwater weighing; DXA: dual-energy X-ray absorptiometry; Cauc: caucasians; SKF: skinfold; Tric: tricipital; Sub: subscapular; M: male; F: female; SPA: single photon absorptiometry; Deut Dilut: deuterium dilution; *R* ^2^: coefficient of determination; RMSE: root of mean square error; CV: coefficient of variation; CCC: concordance correlation coefficient; PE: pure error; FM: fat mass; Prepub: prepubescent; Pub: pubescent; Postpub: postpubescent.

*The authors used a sample aged 8–29 years but recommended the use of the proposed models for children and adolescents aged 8–18 years.

^1^%FM = {(2.747/Db) *‒* (0.727 ∗ TBW/*W*) + (1.146 ∗ BMC/*W*) − 2.053 } ∗ 100, where Db body density; TBW: total body water; BMC: bone mineral content; *W*: body weight [66].
